# Editorial: Predictive and diagnostic approaches for systemic disorders using ocular assessment

**DOI:** 10.3389/fmed.2024.1529861

**Published:** 2024-12-11

**Authors:** Kai Jin, Jiong Zhang, Andrzej Grzybowski

**Affiliations:** ^1^Eye Center, The Second Affiliated Hospital, School of Medicine, Zhejiang University, Hangzhou, China; ^2^Laboratory of Advanced Theranostic Materials and Technology, Ningbo Institute of Materials Technology and Engineering, Chinese Academy of Sciences, Ningbo, Zhejiang, China; ^3^Institute for Research in Ophthalmology, Foundation for Ophthalmology Development, Poznan, Poland

**Keywords:** ocular assessment, systemic diseases, retinal imaging, optical coherence tomography, artificial intelligence

## Introduction

The human eye is not just a window to the soul but also a critical portal through which medical professionals can glean information about a patient's overall health. Advances in technology and research over recent decades have underscored the eye's value as a diagnostic tool for detecting systemic diseases. This editorial explores how ocular assessments can be leveraged for predictive and diagnostic purposes in systemic disorders, underlining the scientific basis and clinical applications of such practices.

## Ocular manifestations of systemic diseases

Systemic diseases often manifest in the eye due to their unique vasculature and neural composition. The retina, for instance, shares similar embryological origins with the brain and is supplied by a rich vascular network. This makes it an ideal site for detecting vascular and neurological changes that reflect systemic conditions. Conditions such as diabetes, hypertension, and autoimmune diseases frequently display characteristic ocular signs, which, when detected early, can facilitate timely interventions.

For example, diabetic retinopathy remains a prominent example of how ophthalmic examinations can reveal the severity and progression of systemic diabetes. Retinal imaging enables the identification of microaneurysms, hemorrhages, and neovascularization, all hallmark features of the disease (1). The presence of these signs not only confirms the diagnosis but can also predict the potential for systemic complications (2).

## Ocular imaging technologies

Advancements in optical coherence tomography (OCT), fundus photography, and retinal angiography have improved the diagnostic capabilities for systemic disorders (3). OCT, with its non-invasive cross-sectional imaging, has been instrumental in assessing macular edema and optic nerve health. It can reveal subtle changes that might correspond to early signs of systemic diseases, including multiple sclerosis and Alzheimer's disease (4). The technique's high resolution enables clinicians to observe changes in the retinal nerve fiber layer (RNFL) thickness, which is crucial in neurological assessments (5).

Artificial intelligence (AI) has also been a game-changer in ocular diagnostics (6). By applying deep learning algorithms to retinal images, researchers have developed predictive models capable of assessing cardiovascular risk factors, such as age, gender, and blood pressure, based solely on retinal scans (7, 8). Such models can transform the way systemic risk stratification is conducted, making assessments more accessible and less invasive.

## Cardiovascular and neurological insights

The retinal microvasculature is often reflective of the broader systemic vascular system. Conditions such as hypertensive retinopathy can reveal not only the presence of high blood pressure but also its duration and impact on vascular health (9). Retinal vascular changes like arteriolar narrowing and arteriovenous nicking are indicative of chronic hypertension and are predictive of an increased risk of stroke (10). Furthermore, studies have shown that monitoring the retinal vessel calibers can serve as an indicator for coronary artery disease, suggesting that ocular assessments could be included as part of a cardiovascular risk assessment protocol (11).

In the realm of neurological disorders, the eye has shown remarkable promise in providing early diagnostic markers. Changes in the optic nerve head and RNFL have been associated with diseases such as Alzheimer's disease and Parkinson's disease. Retinal imaging has demonstrated a thinning of the RNFL in patients with neurodegenerative conditions, correlating with cognitive decline and disease severity (12). This association opens pathways for non-invasive monitoring and early detection, potentially preceding significant brain pathology visible on standard neuroimaging.

## Autoimmune and inflammatory conditions

Autoimmune diseases like systemic lupus erythematosus (SLE) and rheumatoid arthritis (RA) often exhibit ocular manifestations such as uveitis, scleritis, or retinal vasculitis. Ocular assessment not only aids in diagnosing these diseases but can also monitor disease activity and guide treatment (13). Regular eye exams can serve as a practical adjunct to systemic inflammatory markers, providing real-time insight into disease progression.

## Challenges and future directions

While the potential of ocular assessments for systemic disease diagnosis is immense, there are challenges. The integration of eye exams into general medical practice requires enhanced interdisciplinary collaboration between ophthalmologists and other healthcare providers. Additionally, the development and standardization of AI algorithms must ensure reproducibility and fairness across diverse populations to avoid biases that could affect diagnostic accuracy.

Looking ahead, further research should focus on validating ocular biomarkers and integrating these findings into routine clinical practice. Studies exploring the longitudinal relationship between ocular changes and systemic disease outcomes will strengthen the clinical utility of these assessments.

## Conclusion

Ocular assessments hold tremendous promise as a non-invasive, cost-effective means of diagnosing and predicting systemic disorders. As imaging technologies advance and AI becomes more sophisticated, the role of the eye as a diagnostic gateway to broader health assessments will undoubtedly expand. The convergence of ophthalmology with general medicine is an exciting frontier that promises to enhance patient care through earlier detection, more accurate risk stratification, and improved management of systemic diseases.
